# A minimally invasive transpiriformis approach versus the conventional posterolateral approach in total hip arthroplasty for elderly femoral neck fractures: perioperative blood loss and early functional recovery

**DOI:** 10.3389/fsurg.2026.1774770

**Published:** 2026-07-24

**Authors:** Longyun Li, Shaowei Sun, Bobin Fu, Zhixiang Gao, Lijuan Liu, Lifu Wang, Cong Xiao

**Affiliations:** 1 North Sichuan Medical College, Nanchong, China; 2 Department of Orthopedics, The Third Hospital of Mianyang·Sichuan Mental Health Center, Mianyang, China

**Keywords:** femoral neck fracture, functional recovery, minimally invasive transpiriformis approach, perioperative blood loss, total hip arthroplasty

## Abstract

**Background:**

Perioperative blood loss and early recovery are major concerns when elderly patients undergo total hip arthroplasty (THA) for femoral neck fractures. The minimally invasive transpiriformis approach (MIS-TPA), which preserves posterior soft-tissue structures, has recently emerged as an alternative posterior technique. However, its potential benefits over the conventional posterolateral approach (PLA) in reducing blood loss and improving short-term function remain unclear.

**Methods:**

We retrospectively reviewed elderly patients who underwent primary THA for femoral neck fractures at our institution between October 2023 and September 2024. Patients were classified into the MIS-TPA and PLA groups based on the surgical approach. Perioperative blood loss (total, visible, and hidden) was compared between the two groups. Early functional recovery was assessed using the Harris Hip Score (HHS), and prosthetic positioning was evaluated radiographically. Complications during hospitalization and follow-up were recorded.

**Results:**

A total of 138 patients were included in this study (PLA, *n* = 72; MIS-TPA, *n* = 66). Total perioperative blood loss was significantly lower in the MIS-TPA group than in the PLA group (484.60 ± 46.55 mL vs. 953.30 ± 120.68 mL, *P* < 0.001), with a corresponding reduction in hidden blood loss (438.96 ± 46.80 mL vs. 659.85 ± 124.47 mL, *P* < 0.001). Patients in the MIS-TPA group achieved a higher HHS at 1 month postoperatively (79.9 ± 4.7 vs. 73.4 ± 6.0, *P* < 0.001). No significant between-group differences in the HHS were observed at 3, 6, or 12 months. No statistically significant between-group differences were observed in perioperative complications, transfusion requirements, or prosthetic positioning; however, this study was not powered to draw definitive conclusions regarding rare complications.

**Conclusions:**

In this cohort of elderly patients undergoing THA for femoral neck fractures, the MIS-TPA was associated with less perioperative blood loss and a more favorable early recovery profile than the PLA. However, this study was not statistically powered to determine differences in rare postoperative complications.

## 1 Introduction

Total hip arthroplasty (THA) has become a well-established treatment for femoral neck fractures in elderly patients, offering reliable pain relief, restoration of hip function, and improvement in quality of life (1, 2). However, age-related reductions in physiological reserve make this population more vulnerable to perioperative blood loss and delays in early recovery (3). Excessive blood loss may increase transfusion requirements and perioperative complications, whereas limited early mobilization can hinder rehabilitation and compromise functional outcomes (4). Therefore, strategies that reduce perioperative blood loss while facilitating early functional recovery remain clinically important for elderly patients undergoing THA.

The posterolateral approach (PLA) remains one of the most commonly used techniques for THA because it offers reliable exposure, clear anatomical planes, and a standardized operative workflow (5). Nevertheless, the PLA typically involves a more extensive release of the short external rotator muscles. Even with meticulous repair, native soft-tissue tension and stability may not be fully restored, potentially increasing the risk of postoperative dislocation (1%–3%) (6). In addition to its implications for posterior stability, greater posterior muscle injury may increase perioperative blood loss, aggravate postoperative pain, and delay early functional recovery (7). The short external rotator muscles are recognized as key contributors to posterior hip integrity and dynamic stability (8, 9); thus, preserving these structures may promote postoperative recovery.

Over the past decade, minimally invasive THA (MIS-THA) has gained increasing interest as a means of reducing surgical morbidity (10). Over the past decade, a range of minimally invasive THA techniques (e.g., direct anterior, anterolateral, lateral, and modified posterolateral approaches) have been developed to reduce soft-tissue trauma and facilitate early recovery, although each approach entails distinct trade-offs in exposure, soft-tissue handling, and learning curve (11, 12). Building on the concept of posterior muscle preservation (13, 14), Roger and Hill introduced the MIS-TPA, in which only the piriformis tendon is released, while the remaining short external rotators are preserved, and reported lower transfusion rates and faster postoperative recovery in their early experience (15). Furthermore, our group previously reported that the MIS-TPA was associated with a milder postoperative inflammatory response than the PLA, reflected by lower levels of muscle injury and inflammatory markers (16).

Despite these promising findings, it remains unclear whether the MIS-TPA yields clinically meaningful reductions in perioperative blood loss and improvements in early postoperative function, particularly in elderly patients undergoing THA for femoral neck fractures. Therefore, the purpose of this study is to compare perioperative blood loss and early functional outcomes between the MIS-TPA and the conventional PLA in elderly patients.

## 2 Methods

### 2.1 Study design and participants

This was a single-center retrospective cohort study conducted at our institution between October 2023 and September 2024. We retrospectively reviewed elderly patients with femoral neck fractures who underwent primary THA during the study period. Patients were screened according to the predefined inclusion and exclusion criteria. The inclusion criteria were as follows: (1) age >60 years; (2) radiographically confirmed unilateral displaced femoral neck fracture; (3) treatment with primary THA; (4) surgery performed using either the MIS-TPA or the conventional PLA. Patients were excluded if they had the following: (1) open or old fractures; (2) pathological fractures; (3) multiple trauma or concomitant fractures at other sites; (4) a history of thromboembolic events prior to surgery, including deep vein thrombosis, cerebral infarction, or pulmonary embolism; (5) discontinuation of anticoagulant therapy for less than 1 week before surgery; (6) known bleeding disorders or hematologic diseases; or (7) incomplete follow-up data at 1 year postoperatively. Patients were classified into the MIS-TPA and PLA groups according to the surgical approach actually used in routine clinical practice; no randomization was involved. Ethical approval was obtained from the institutional ethics committee (No. 2023-56).

### 2.2 Surgical procedures and perioperative management

All procedures were completed by a consistent surgical team comprising one senior surgeon and two attending surgeons. The senior surgeon was experienced in both approaches and had performed more than 100 THAs using each technique before the study period. Perioperative management was applied uniformly to all patients, including routine antibiotic prophylaxis and thromboprophylaxis. Postoperatively, all patients followed the same rehabilitation protocol, beginning with early range-of-motion exercises and subsequently progressing to structured muscle-strengthening training.

### 2.3 Surgical approach

The minimally invasive transpiriformis approach: Patients were positioned in the lateral decubitus position. An approximately 8–10 cm incision was made over the greater trochanter. After incising the skin and subcutaneous tissue, the gluteus maximus was bluntly separated along the direction of its fibers to expose the piriformis muscle. The piriformis tendon was released at its insertion, while the remaining short external rotator muscles—including the obturator internus and superior gemellus—were preserved. The acetabulum was prepared and the component was implanted using standard techniques, with supplemental screw fixation when required. Femoral components were implanted based on preoperative templating and intraoperative assessment, followed by hip reduction with external rotation. Stability was evaluated through a full range-of-motion examination. After thorough irrigation, the capsule was repaired and the piriformis tendon was reconstructed. No postoperative drainage was used, and the wound was closed with subcuticular sutures. Detailed technical aspects have been described previously (16).

The conventional posterolateral approach: The PLA procedure was performed in the same lateral decubitus position. A skin incision of approximately 14 cm was made, centered over the greater trochanter. After the skin and subcutaneous tissue were incised, the gluteus maximus was split, followed by release of the short external rotators and opening of the posterior capsule. Subsequent steps, including hip dislocation, femoral neck osteotomy, and implantation of the acetabular and femoral components, were performed in the same manner as in the MIS-TPA group. In addition, a postoperative drainage tube was routinely placed and removed once the drainage volume was <50 mL within 24 h.

### 2.4 Outcome measures

#### 2.4.1 Baseline characteristics and perioperative variables

Baseline patient characteristics were documented at admission, including age, sex, body mass index (BMI), and American Society of Anesthesiologists (ASA) grade. Operative variables included incision length and operative time, time to surgery, and length of hospital stay.

#### 2.4.2 Assessment of perioperative blood loss

Perioperative blood loss was calculated using established formulas and reported as total blood loss (TBL), visible blood loss (VBL), and hidden blood loss (HBL). Patient blood volume was estimated using the Nadler formula (17), as follows:

$$\begin{array}{rl} \mathrm{PBV}\;(L)\;= & \;\mathrm{height}\;(m{)}^{3}\;\;\times \;0.367\;+\;\mathrm{weight}\;(\mathrm{kg})\;\;\times \;0.032\\ & +\;0.604\;\mathrm{for}\;\mathrm{men}\\ & \;\mathrm{PBV}\;(L)\;=\;\mathrm{height}\;(m{)}^{3}\;\;\times \;0.356\;+\;\mathrm{weight}\;(\mathrm{kg})\;\;\times \;0.033\\ & +\;0.183\;\mathrm{for}\;\mathrm{women} \end{array}$$

Total blood loss was then derived using the Gross formula (18):

$$\mathrm{TBL}\;(\mathrm{mL})\;=\;\mathrm{PBV}\;(L)\;\;\times \;(\mathrm{Hc}t_{\mathrm{pre}}-\;\mathrm{Hc}t_{\mathrm{post}})\;/\;\mathrm{Hc}t_{\mathrm{mean}}\;\;\times \;1,000$$

where Hct_pos_t refers to the hematocrit measured on postoperative day 3, and Hct_mean_ represents the mean of preoperative and postoperative hematocrit values.

VBL was estimated based on intraoperative suction volume and the calculated blood content of surgical sponges after adjustment for irrigation fluid; postoperative drainage volume was included when applicable. Blood loss from a fully saturated sponge was standardized at approximately 20 mL. HBL was calculated as follows:

HBL(mL)=TBL(mL)+transfusionvolume(mL)-VBL(mL)

For reference, one unit of packed red blood cells was defined as 200 mL. Allogeneic transfusion was administered when hemoglobin levels were <70 g/L or hematocrit was <25%. In patients older than 65 years or with cardiovascular/respiratory comorbidities, transfusion was considered at hemoglobin <80 g/L when acute anemia was clinically evident.

In this femoral neck fracture cohort, calculated blood loss values were interpreted as estimates of perioperative hematologic loss rather than purely surgery-specific blood loss. Because both groups had the same underlying pathology and were assessed using the same calculation method, these values were considered appropriate for relative between-group comparison.

#### 2.4.3 Assessment of Hip function and muscle injury

Hip function was assessed using the HHS at 1, 3, 6, and 12 months postoperatively. Early functional recovery was further characterized by the time to first ambulation and the time required to achieve independent stair climbing. To evaluate perioperative muscle injury and inflammatory response, serum creatine kinase (CK), myoglobin, and C-reactive protein (CRP) levels were measured preoperatively and on postoperative days 1 and 3.

#### 2.4.4 Radiographic evaluation and complications

Standard postoperative radiographs and computed tomography scans were obtained to evaluate prosthetic positioning, including acetabular inclination and anteversion angles. Postoperative complications were systematically assessed during hospitalization and throughout the 12-month postoperative follow-up period, including hip dislocation, surgical site infection, periprosthetic joint infection, periprosthetic fracture, deep vein thrombosis, and other major medical complications.

### 2.5 Statistical analysis

Data were entered in Excel and analyzed in SPSS Statistics (version 27.0; IBM Corp., Armonk, NY, USA). Variable distribution was assessed prior to group comparisons. Normally distributed continuous variables were reported as mean ± standard deviation and were compared using the independent-samples *t*-test; non-normally distributed data were reported as median (interquartile range) and were compared using the Mann–Whitney U test. The *χ*^2^ test or Fisher's exact test was applied for categorical variables. Two-sided *P*-values < 0.05 were considered statistically significant.

## 3 Results

### 3.1 Patient characteristics

During the study period, a consecutive series of 160 elderly patients who met the inclusion criteria was identified. After application of the predefined exclusion criteria, 22 patients were excluded because of the following reasons: 1 had an old femoral neck fracture, 1 had a concomitant proximal humeral fracture, and 20 had incomplete 12-month follow-up data. As a result, 138 patients were included in the final analysis (MIS-TPA, *n* = 66; PLA, *n* = 72). No significant between-group differences were found in baseline characteristics, including age, sex, body mass index, and ASA grade (*P* > 0.05) (Table 1).

**Table 1:** Demographic data of the patients.

Characteristics	MIS-TPA group (*n* = 66)	PLA group (*n* = 72)	*P*-value
Sex (M/F) (no. of patients, %)	25 (37.9)/41 (62.1)	29 (40.3)/43 (59.7)	0.78
Age (year)	77.45 ± 8.67	75.86 ± 7.63	0.25
Height (m)	1.56 ± 0.09	1.58 ± 0.08	0.11
Weight (kg)	56.77 ± 9.11	57.24 ± 8.36	0.76
BMI (kg/m^2^)	23.34 ± 3.91	22.89 ± 3.57	0.48
ASA score (no. of patients, %)			0.72
Ⅰ	7 (10.6)	11 (15.3)	
Ⅱ	30 (45.5)	27 (37.5)	
Ⅲ	21 (31.8)	23 (31.9)	
Ⅳ	8 (12.1)	11 (15.3)	

ASA score, American Society of Anesthesiologists Classification score; BMI, body mass index.

### 3.2 Perioperative blood loss and surgical parameters

Patients in the MIS-TPA group had a substantially lower VBL than those in the PLA group (45.64 ± 10.35 mL vs. 293.46 ± 39.09 mL, *P* < 0.001). Because no postoperative drainage was used in the MIS-TPA group, postoperative drainage volume was recorded only in the PLA group, with a mean volume of 101.81 ± 35.45 mL. TBL was also significantly reduced in the MIS-TPA group (484.60 ± 46.55 mL vs. 953.30 ± 120.68 mL, *P* < 0.001), with a similar reduction observed in HBL (438.96 ± 46.80 mL vs. 659.85 ± 124.47 mL, *P* < 0.001). The proportion of patients receiving allogeneic blood transfusion was lower in the MIS-TPA group (two patients) than in the PLA group (seven patients), although the difference was not statistically significant (*P* = 0.11). Incision length (8.99 ± 0.68 cm vs. 15.10 ± 1.20 cm, *P* < 0.001) and operative time (63.95 ± 9.15 min vs. 86.28 ± 13.25 min, *P* < 0.001) were both shorter in the MIS-TPA group. Hospital stay was significantly shorter in the MIS-TPA group than in the PLA group (6.03 ± 2.00 days vs. 11.28 ± 2.68 days, *P* < 0.001) (Table 2).

**Table 2:** Perioperative blood loss, surgery-related data, hip function, and muscle injury.

Variables	MIS-TPA group	PLA group	*P-*value
Surgery-related data			
Incision length (cm)	8.99 ± 0.68	15.10 ± 1.20	<0.001
Operation time (min)	63.95 ± 9.15	86.28 ± 13.25	<0.001
Hospital stay (days)	6.03 ± 2.00	11.28 ± 2.68	<0.001
Time to operation(h)	20.03 ± 6.60	22.36 ± 8.50	0.08
Perioperative blood loss			
Total blood loss (TBL, mL)	484.60 ± 46.55	953.30 ± 120.68	<0.001
Hidden blood loss (HBL, mL)	438.96 ± 46.80	659.85 ± 124.47	<0.001
Visible blood loss (VBL, mL)	45.64 ± 10.35	293.46 ± 39.09	<0.001
Number of patients requiring blood transfusion (n, %)	2 (3.03%)	7（9.72%）	0.11
Postoperative function			
Time to bedside ambulation (h)	4.20 ± 1.60	19.74 ± 4.41	＜0.001
Time to using stairs independently (days)	1.64 ± 0.62	3.49 ± 0.81	＜0.001
HHS score			
1 month postoperatively	79.91 ± 4.65	73.43 ± 5.95	<0.001
3 months postoperatively	86.02 ± 4.18	84.75 ± 3.74	0.06
6 months postoperatively	90.03 ± 2.99	89.13 ± 2.60	0.06
12 months postoperatively	90.95 ± 2.84	90.42 ± 2.47	0.24
CK(U/L)			
1st day preoperative	84.98 ± 35.96	87.17 ± 33.71	0.71
1st day postoperative	537.42 ± 204.23	644.98 ± 39.64	<0.05
3rd day postoperative	401.91 ± 130.61	552.94 ± 96.11	<0.05
Myoglobin (ng/mL)			
1st day preoperative	62.91 ± 21.90	63.55 ± 24.70	0.87
1st day postoperative	470.00 ± 219.56	590.72 ± 27.27	<0.05
3rd day postoperative	171.37 ± 90.19	219.88 ± 79.26	<0.05
CRP(mg/L)			
1st day preoperative	12.11 ± 5.89	13.03 ± 4.47	0.30
1st day postoperative	60.94 ± 15.64	70.00 ± 12.20	<0.05
3rd day postoperative	91.66 ± 29.74	100.80 ± 11.84	<0.05

### 3.3 Radiographic evaluation

**Figure 1:**
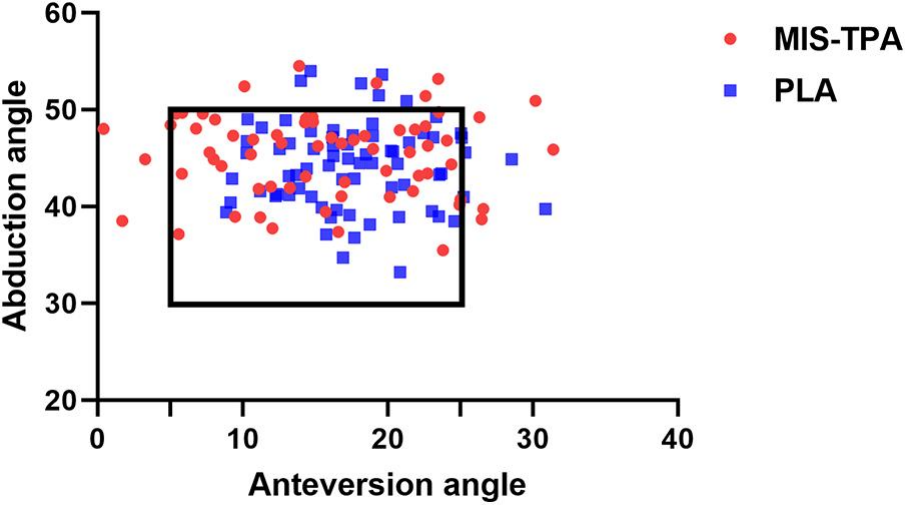
Acetabular component positioning in the two groups of patients. The *x*-axis represents the anteversion angle of the acetabular prosthesis, while the *y*-axis represents the abduction angle of the acetabular prostheses. The boxes in the figure represent the Lewinnek “Safe zone.”

Postoperative radiographs showed comparable acetabular component positioning between the two groups. No significant differences were observed in acetabular cup inclination (45.30 ± 4.36° vs. 44.21 ± 4.39°, *P* = 0.146) or anteversion angles (15.74 ± 7.35° vs. 17.68 ± 4.80°, *P* = 0.066) between the groups. Further evaluation based on the Lewinnek safe zone (inclination, 30°–50°; anteversion, 5°–25°) showed no significant differences in the proportion of acceptable acetabular cup positioning. Acceptable anteversion was achieved in 87.9% of patients in the MIS-TPA group and 91.7% in the PLA group, while acceptable inclination was observed in 93.9% and 91.7%, respectively (Figure 1).

### 3.4 Early functional recovery and muscle injury markers

Early functional recovery outcomes are presented in Table 2. Bedside ambulation occurred significantly earlier in the MIS-TPA group than in the PLA group (4.20 ± 1.60 h vs. 19.74 ± 4.41 h, *P* < 0.001), and independent stair climbing was also achieved sooner (1.64 ± 0.62 days vs. 3.49 ± 0.81 days, *P* < 0.001). The MIS-TPA group had a higher HHS at 1 month postoperatively (79.91 ± 4.65 vs. 73.43 ± 5.95, *P* < 0.001); however, this difference was no longer significant at 3, 6, or 12 months. Preoperative CK, myoglobin, and CRP levels were similar between the groups; however, on postoperative days 1 and 3, these markers were lower in the MIS-TPA group than in the PLA group (*P* < 0.05).

### 3.5 Perioperative complications

No major complications were recorded in either group during hospitalization or follow-up, including wound-related, medical, and prosthesis-related events. One hip dislocation (1.39%) occurred in the PLA group, whereas none occurred in the MIS-TPA group. Lower-extremity deep vein thrombosis was identified in one patient (1.51%) in the MIS-TPA group and six patients (8.33%) in the PLA group (*P* = 0.07) (Table 3). No statistically significant between-group differences were observed in perioperative complications. However, this study was not powered to draw definitive conclusions regarding rare complications such as dislocation and deep vein thrombosis.

**Table 3:** Perioperative complications

Complications	MIS-TPA group [*n* (%)]	PLA group [*n* (%)]	*P*-value
Incision infection/subcutaneous hematoma	0 (0)	0 (0)	
Pressure ulcer	0 (0)	0 (0)	
Pulmonary infection	0 (0)	0 (0)	
Cerebral infarction	0 (0)	0 (0)	
Prosthesis loosening/periprosthetic fracture	0 (0)	0 (0)	
Periprosthetic joint infection	0 (0)	0 (0)	
Prosthesis dislocation	0 (0)	1 (1.39)	
Deep vein thrombosis	1 (1.51)	6 (8.33)	0.07

## 4 Discussion

Our findings indicate that, among elderly patients undergoing THA for femoral neck fractures, the MIS-TPA was associated with lower perioperative blood loss and more favorable early recovery than the conventional PLA. No statistically significant between-group differences were observed in prosthetic positioning. Likewise, no statistically significant between-group differences were observed in perioperative complications; however, this study was not powered to draw definitive conclusions regarding rare complications.

Posterior muscle–preserving strategies in THA have been reported previously; however, substantial variability exists among different modified techniques in terms of posterior soft-tissue handling. Khan et al. reported favorable clinical outcomes in a cohort of 100 patients treated with a modified traditional posterior approach (13). Kim et al. described a related technique that preserved the piriformis muscle and part of the quadratus femoris, achieving satisfactory results without the use of specialized acetabular instruments (14). Notably, according to conventional definitions that rely primarily on incision length, these techniques are not uniformly classified as “minimally invasive” (incision length ≤ 10 cm) (19). Nevertheless, the favorable perioperative outcomes reported with these modified approaches suggest that defining “minimally invasive” procedures solely by incision length may not adequately reflect the true extent of surgical trauma, and that perioperative outcomes may be more closely related to the manner in which posterior soft tissues are managed. On this basis, Roger and Hill further proposed and reported the MIS-TPA technique, in which only the piriformis tendon is released at its insertion, while the remaining short external rotators are preserved. This approach minimizes direct injury and excessive traction to deep muscle fibers, structurally maintains the integrity of the joint capsule and muscle units, and has been associated with favorable early clinical outcomes (15). By preserving a familiar posterior operative corridor without the need for specialized instruments, the MIS-TPA emphasizes protection of posterior soft-tissue structures, which may partly explain the lower blood loss and faster early functional recovery observed in patients in the present study.

Perioperative blood loss is an important determinant of recovery after THA, particularly in elderly patients with limited cardiopulmonary reserve who are more vulnerable to postoperative anemia and intravascular volume fluctuations (20). In the present study, both total blood loss and hidden blood loss were lower in the MIS-TPA group than in the PLA group, suggesting a lower perioperative hematologic burden with this approach. Reduced perioperative blood loss may help preserve postoperative hemoglobin levels and tissue oxygenation, thereby mitigating early fatigue and anemia-related symptoms (21). These findings should nevertheless be interpreted in the context of the drainage protocol used in the PLA group. Drain use may have influenced the postoperative presentation of blood loss, particularly the measurable visible component, and should therefore be regarded as a potential methodological confounder (22). In addition, perioperative blood loss is likely influenced not only by the extent of surgical exposure, but also by the degree of soft-tissue injury and the subsequent inflammatory response (23, 24). In this study, postoperative CK, myoglobin, and CRP levels were lower in the MIS-TPA group, suggesting less periarticular soft-tissue injury and supporting the muscle-sparing nature of this approach. These physiological differences may have contributed to the lower hematologic burden observed in the MIS-TPA group and to a more favorable condition for early recovery.

Multiple factors influence early recovery after THA, including prosthetic positioning and perioperative surgical stress. In the present study, the MIS-TPA group had a higher HHS at 1 month. The between-group difference was 6.5 points, which was statistically significant. However, statistical significance does not necessarily indicate a patient-perceived clinically meaningful benefit. Singh et al. reported clinically important improvement thresholds for the HHS of approximately 15.9–18 points after primary THA (25). Therefore, although the 1-month HHS difference suggests a statistically better early functional score in the MIS-TPA group, it did not reach the commonly cited clinically important threshold reported in the THA literature. Moreover, an minimal clinically important difference (MCID) specific to elderly patients undergoing THA for femoral neck fractures has not been established. This finding should therefore be interpreted with caution rather than as definitive evidence of a clinically meaningful early recovery benefit. However, because no significant between-group difference was observed after 3 months, this finding should be interpreted as an early recovery advantage rather than sustained long-term functional superiority. In addition, statistical significance does not necessarily indicate a patient-perceived clinically meaningful benefit, and the clinical relevance of the early HHS difference should therefore be interpreted cautiously.

Prior studies have shown that preservation of the short external rotators during posterior-approach THA is associated with faster recovery and better early hip function (26, 27). In the present cohort, reduced posterior soft-tissue injury, together with lower perioperative blood loss, may have facilitated earlier mobilization and contributed to the more favorable early functional profile observed in the MIS-TPA group. However, the marked difference in time to first ambulation should be interpreted cautiously, because a postoperative closed-suction drain was routinely used in the PLA group but not in the MIS-TPA group. Drain tethering itself may have restricted early mobilization and therefore represents a plausible independent contributor to delayed ambulation, distinct from the surgical approach and soft-tissue injury. This may also partly explain the shorter hospital stay in the MIS-TPA group. Previous evidence further indicates that posterior muscle preservation is associated with lower early postoperative pain and reduced muscle injury and inflammatory responses (28, 29). As healing progresses, the relative contribution of these perioperative factors to functional outcome is likely to diminish, which may explain the attenuation of between-group differences over time.

Not all minimally invasive techniques yield consistent perioperative or early recovery benefits. Meng et al. reported greater perioperative blood loss with the SuperPath approach than with the conventional posterolateral approach in staged THA, in contrast to the present findings (30). This discrepancy suggests that perioperative performance is influenced not only by incision length, but also by surgical workflow, instrumentation, and operative efficiency. During the learning curve, the constrained operative corridor and use of specialized cannulas in the SuperPath technique may increase procedural complexity and operative time, potentially offsetting the benefits of reduced soft-tissue exposure (31). These observations underscore the heterogeneity of minimally invasive techniques and suggest that outcomes may depend more on soft-tissue handling and procedural control than on incision length alone. In the present study, all procedures were performed by a consistent surgical team with substantial experience in both THA and minimally invasive techniques, which may have reduced technique-related variability and contributed to the observed perioperative consistency.

Several limitations should be considered in this study. First, the retrospective, non-randomized design introduces an inherent risk of selection bias, and residual confounding cannot be entirely excluded despite generally comparable baseline characteristics. The relatively small sample size may also limit external validity, particularly for infrequent outcomes. Accordingly, the study was not statistically powered to draw definitive conclusions regarding rare complications such as dislocation and deep vein thrombosis, and these findings should therefore be interpreted only as descriptive findings. Second, the relatively low BMI of the study population reflects regional characteristics and may restrict the generalizability of the findings to populations with higher BMI, in whom achieving a short incision and minimally invasive exposure may be more technically challenging. Importantly, minimally invasive surgery should be interpreted not only by incision length but also by the degree of soft-tissue preservation. Third, an additional methodological consideration relates to blood-loss estimation. Because patients with femoral neck fractures may sustain hidden blood loss before surgery, the calculated blood loss values should be interpreted as estimates of perioperative hematologic loss rather than purely surgery-specific blood loss. Nevertheless, the use of the same calculation method in two clinically comparable groups still permits a relative between-group comparison. In addition, the routine use of drainage in the PLA group may have influenced the postoperative presentation of blood loss and should be regarded as a potential methodological confounder. Moreover, the same drainage protocol may also have confounded early mobilization outcomes, because patients with a closed-suction drain may have been less able or less willing to ambulate early. Therefore, the difference in time to first ambulation should not be attributed solely to the surgical approach. Fourth, perioperative management and rehabilitation protocols were standardized within our institution, which may limit comparability with other centers. Although the MIS-TPA group showed a more favorable early functional profile, no significant between-group difference in the HHS was observed after 3 months. Therefore, this finding should be interpreted as an early recovery advantage rather than sustained long-term functional superiority. Although the 1-month HHS difference was statistically significant, it was below the clinically important improvement threshold of approximately 15.9–18 points reported for the HHS after primary THA. Because no fracture-specific MCID for the HHS has been established in elderly patients undergoing THA for femoral neck fractures, the clinical relevance of this early HHS difference should be interpreted cautiously.

Finally, this study primarily evaluated early postoperative recovery, and long-term functional outcomes, as well as more detailed radiographic assessments, were not systematically examined. Larger, multicenter prospective studies with longer follow-up are needed to confirm these findings.

## 5 Conclusion

In this cohort of elderly patients undergoing total hip arthroplasty for femoral neck fractures, the MIS-TPA was associated with less perioperative blood loss and more favorable early functional recovery than the PLA. However, this study was not statistically powered to draw definitive conclusions regarding rare postoperative complications.

## Data Availability

The raw data supporting the conclusions of this article will be made available by the authors, without undue reservation.
